# Beneficial Effects of Photoperiod Lengthening on Sleep Characteristics and Mechanical Hyperalgesia in Injured Rats

**DOI:** 10.1523/ENEURO.0433-23.2023

**Published:** 2024-03-01

**Authors:** T. Vanneau, M. Quiquempoix, M.-C. Erkel, C. Drogou, A. Trignol, F. Sauvet, D. Léger, D. Gomez-Merino, M. Chennaoui

**Affiliations:** ^1^French Armed Forces Biomedical Research Institute (IRBA), Brétigny-sur-Orge 91223, France; ^2^VIFASOM (URP 7330 Vigilance, Fatigue, Sommeil et Santé Publique), Université Paris Cité, Paris 75001, France; ^3^APHP, APHP-Centre Université de Paris, Hôtel-Dieu, Centre du Sommeil et de la Vigilance, Paris 75001, France

**Keywords:** biomarkers, brain, hyperalgesia, muscle injury, photoperiod, sleep regulation

## Abstract

Sleep and muscle injury-related pain are in negative relationship, and sleep extension may be a favorable countermeasure. In response to muscle injury, an adaptive sleep response has been described in rats, characterized by an increase in total sleep time (TST) and nonrapid eye movement (NREM) sleep. This study examined the effects of photoperiod lengthening (a model of sleep prolongation in rats) on the sleep characteristics of muscle-injured rats and whether this lengthening could benefit injury-induced mechanical hyperalgesia using the Von Frey test. Switching from the conventional 12:12 light/dark (LD) photoperiod (light on: 08:00–20:00) to LD 16:8 (light extended to 24:00) gives rats an extra window of sleep. Our results show higher TST and NREM sleep times in LD 16:8 versus LD 12:12 injured rats during 4 h of light lengthening for 7 d postinjury, showing the efficiency of photoperiod lengthening to increase sleep time in injured rats. In addition, a cumulative effect with the adaptive sleep response to muscle injury occurred with higher TST and NREM sleep times in LD 16:8 injured versus noninjured rats during the dark period, reflecting the high need for sleep after the injury. Greater stability and higher relative delta power of NREM sleep during the extended light period were also observed in injured rats. Finally, the extended photoperiod limits the muscle injury-induced mechanical hyperalgesia for 13 d and allows faster recovery of the baseline mechanical threshold. This is associated with reduced pro-inflammatory cytokines levels in the hippocampus, a brain structure involved in pain processing.

## Significance Statement

Our results demonstrate the efficiency of photoperiod lengthening to induce sleep extension in rats after muscle injury. Furthermore, there is a cumulative effect of photoperiod lengthening *plus* adaptive sleep response to muscle injury on sleep time, suggesting a significant need for sleep in injured rats. In addition, the photoperiod lengthening limits muscle injury-induced mechanical hyperalgesia and allows faster recovery of baseline paw withdrawal threshold, associated with a decrease in pro-inflammatory cytokines in the hippocampus. These results suggest that photoperiod lengthening could potentially be used as a nonpharmacological treatment for mechanical hyperalgesia after muscle injury.

## Introduction

The interactions between sleep and pain have been widely described for their adverse effects. Several studies have shown that a vicious circle between pain and sleep develops during chronic pain (Pilowsky et al., 1985; Call-Schmidt and Richardson, 2003; Brennan and Lieberman, 2009; Faraut et al., 2015; Harrison et al., 2016; Rosseland et al., 2018): pain decreases sleep quality (Power et al., 2005; Foley et al., 2007) and poor sleep quality is often associated with increased pain intensity (Kundermann et al., 2004; Haack and Mullington, 2005; Simpson et al., 2018). Restorative sleep (i.e., quantitative improvements in sleep) has been shown to reduce pain sensitivity, playing a major role in the management of chronic pain (Roehrs et al., 2012). Recently, Simonelli et al. (2019) showed that sleep extension increases pain tolerance but does not affect the pain threshold in normally sleeping individuals. However, the effects of sleep extension in a painful context, such as a muscle injury, have never been evaluated.

Muscle injuries are common in athlete and military populations (Kaufman et al., 2000; Ekstrand et al., 2011). Our recent study showed the effects of skeletal muscle injury on sleep parameters in rodents (Vanneau et al., 2023). This study describes an adaptive response of sleep with an acute increase in NREM sleep time during the preferred period of activity (i.e., during the dark period) at 48 and 72 h postinjury associated with an increase in sleep stability (Vanneau et al., 2023). Such results suggest an increased need for sleep in response to muscle injury. We therefore questioned whether potentiating this adaptive response by providing larger sleep windows might be beneficial for mechanical hyperalgesia associated with the muscle injury.

In humans, this is possible by increasing the time spent in bed, leading to an increase in total sleep time and better pain tolerance (Chennaoui et al., 2016; Simonelli et al., 2019). For rodents, which are nocturnal animals, increasing the duration of the light period from 12 h of light per day to 16 h adds 4 h of high sleep propensity and has been described as inducing sleep extension on healthy rats (Sauvet et al., 2018; Vanneau et al., 2021). Yet, the feasibility of sleep extension in injured animals and its effects on mechanical hyperalgesia induced by the muscle injury have not been described yet.

To this end, we initially characterize the sleep structure of muscle-injured animals submitted to either conventional light/dark (LD) 12:12 (lights on: 08:00–20:00) or extended LD 16:8 photoperiod (light extended to 24:00). Our findings demonstrate the efficiency of photoperiod lengthening to induce sleep extension in rats after muscle injury. Furthermore, a cumulative effect of the extended light period *plus* the adaptive sleep response to muscle injury was observed, with higher NREM sleep time in LD 16:8 injured animals. In addition, NREM sleep of LD 16:8 injured animals was associated with both, higher stability, and higher relative delta power during the extended light period. On the other hand, photoperiod lengthening limits the muscle injury-induced mechanical hyperalgesia and allows faster recovery of the baseline paw withdrawal threshold reflecting pain sensitivity, measured through a Von Frey test. These results demonstrate for the first time the potential benefits of photoperiod lengthening as a nonpharmacological intervention for pain management after muscle injury in rats.

## Materials and Methods

### Experimental design

The experiment was performed using 32 male Wistar rats (Charles River Laboratories) divided into two main groups: a group (*n* = 16) for sleep analysis (S-) with implanted animals to record the EEG/EMG and a group (*n* = 16) for pain analysis (P-) with nonimplanted animals. After 1 week of habituation, animals were housed four per cage (612 × 435 × 216 mm) on a 12 h light and 12 h dark photoperiod (LD 12:12; lights from 08:00 to 20:00) with food and water available ad libitum in temperature (21°C ± 2°C) and ventilated (ACH 70) controlled environment. Animals for sleep analysis were implanted with telemetry transmitters to record EEG/EMG (HD-X02, Data Sciences International). These transmitters consist of two bipolar electrodes and transmit signals via Wi-Fi to a recording plate located under the animal's cage. After surgery, animals were individually housed in a ventilated cabinet (BIO-C36, Tecniplast) and had a 10 d recovery period. Transmitter recordings started for 3 d (baseline: Day −3, Day −2, Day −1) before cardiotoxin injection and photoperiod change.

**Figure 1.**
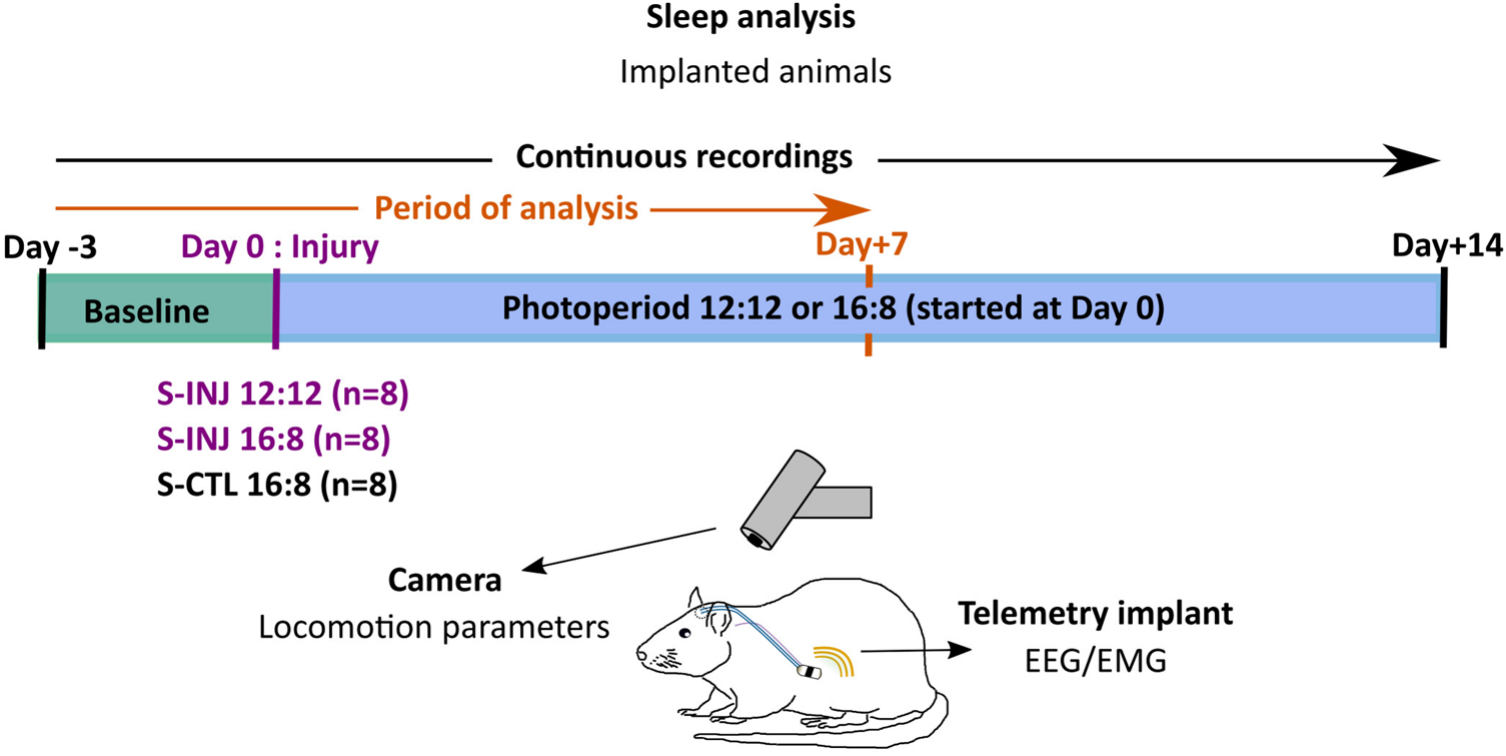
Schematic representation of the experimental procedure. Animals for sleep analysis were implanted with telemetry to record EEG/EMG continuously from Day −3 to Day +14 (analysis were done between Day −3 and Day +7) and were filmed continuously to measure their locomotion parameters. They were separated into two groups (*n* = 8 per group): sleep-control (S-CTL) light/dark (LD) 16:8 (hours of light:hours of dark) and S-INJ LD 16:8. The S-injured (INJ) LD 12:12 is derived from our previous publication (Vanneau et al., 2023). Muscle injury (cardiotoxin injection in the TA) and photoperiod change were made at Day 0 at 08:00. The baseline period (Day −3 to Day −1) was under a LD 12:12 photoperiod for all animals.

Animals for sleep analysis (S-) were separated into two groups (*n* = 8 per group): an injured (INJ) and a control (CTL) group under an LD 16:8 photoperiod (S-INJ 16:8 and S-CTL 16:8). Injured animals received a 400 µl (10 µM) injection of cardiotoxin (Latoxan) in the right tibialis anterior (TA) muscle (without opening the skin) under a 10 min vetflurane (1–2%) anesthetic and a single subcutaneous injection of buprenorphine (0.05 mg/kg) for analgesia, all at 08:00 at Day 0. To control for the potential effects of isoflurane anesthesia on sleep–wake architecture (Jang et al., 2010), we also anesthetized control animals for the same duration as the injured animals (∼10 min.). The S-CTL 16:8 animals were also anesthetized during 10 min under vetflurane (1–2%) to avoid the potential effects of anesthesia on sleep–wake architecture control anesthetic effects on wake/sleep parameters. The photoperiod changes were made right after the cardiotoxin injection (Day 0–08:00). In Figure 1, the S-INJ LD 12:12 group, derived from our previously published data (Vanneau et al., 2023), is represented and its data reused.

The two other groups of animals were considered for pain analysis (P-), following the same procedure but without telemetry EEG/EMG surgical implantation: one group (*n* = 8) of injured 12:12 animals (P-INJ 12:12) and one group (*n* = 8) of injured 16:8 animals (P-INJ 16:8). Sleep-animals and Pain-animals were killed 14 d after the injection, and we collected right and left TA muscles and two brain areas, the hippocampus and the frontal cortex, for Pain-animals. The study was approved by the institutional animal ethics committee (Protocol DAP 2018-11).

### EEG/EMG signal analyses

Sleep-animals were implanted with transmitters for the recording of EEG/EMG (HD-X02, Data Sciences International). The procedure is described in our recent study (Vanneau et al., 2021). Manual classification of 10 s epoch as wake, NREM sleep, and REM sleep have been done according to McShane et al. (2010) principles. The total sleep time (TST, NREM + REM sleep time), the total NREM sleep time, and the total REM sleep time were evaluated for each hour, and we calculated daily average. To evaluate the sleep stability, for each hour, we noted the duration of each NREM and REM sleep periods and grouped them into the following categories, [10–40 s], [50–160 s], [>170 s], and were divided by the time spent in the corresponding state (NREM/REM) for the corresponding hour. Relative delta power (0.25–4.0 Hz power/0.25–40 Hz power) was calculated by applying a fast Fourier transform with pwelch method (4 s window with 50% overlap on filtrated signals between 0.1 and 40 Hz) on all NREM periods per hour (all 10 s periods were grouped together). Same as for relative theta (4.25–8.0 Hz) power during REM sleep. Spindles were detected using the approach described in Uygun et al. (2019).

### Video recording analyses

Video recordings (30 FPS, 1,920 × 1,080 px) were made with Axis P1365 Mk II camera (Axis Communications) using Raytec infrared illuminators (VAR2-IA-1C, Raytec). Analysis of distance covered and the percentage of body in motion were performed with EthoVision (EthoVision XT 15.0, Noldus).

### Assessment of muscle injury-induced mechanical hyperalgesia

Static mechanical threshold was measured as paw withdrawal threshold using an electronic Von Frey 5 test (BIO-EVF5, Bioseb) with restraining cages (BIO-PVF, Bioseb). The test is described in Ferrier et al. (2016). In short, we manually apply increasing force under the animal's paw using a plastic cone, with electronic feedback to help us apply increasing pressure. As soon as the animal removes its paw, the corresponding force is automatically recorded and corresponds to the mechanical sensitivity threshold. During each test, we performed two measurements per paws. For the week of habituation, animals were put in the restraining cage for 1–2 h per day and gently stimulated with the plastic cone. On the last 2 d of habituation, the Von Frey test was performed, and measurements are recorded on the first day of baseline (Day −3 before injection). A test is then performed every day at 16:00 from Day −3 to Day +13, except for the first 3 d after the injection.

### Killing and histological and biochemical analyses

Rats were killed by decapitation, the TA muscles were harvested and frozen by dipping them in isopentane (−100°C) and 8 µm cryocuts were performed on Cryostat (CryoStar NX70, Epredia). Wheat germ agglutinin (WGA) immunostainings were made by a first 1 h blockade with Emerald antibody (#936B-08 Sigma-Aldrich). Then muscle cuts were incubated with a primary antibody [(1/1,000] WGA #ab11575, Abcam] overnight at 4°C. We used a mounting medium with Dapi (#ab104139, Abcam). Slides were then scanned (10×, Axioscan Z1, Zeiss) and injured area was calculated manually on FIJI (ImageJ) as the total area with centronuclei muscle fibers/total muscle area. Hemalun–Phloxin–Safran (HPS) coloration was made by dipping slides in Hemalun (for 100 ml: 0.2 g hematein, 5 g potassium alum, 2 ml of acetic acid, 98 ml of distilled water), Phloxin (0.5 g/100 ml), and Safran (2 g/100 ml) interspersed with washings in water and alcohol (70, 95, 100%). The frontal cortex and hippocampus were dissected immediately after the sacrifice on a cold plate and deep-frozen in liquid nitrogen. Brain areas were homogenized in 2 ml Precellys CK14 tubes containing 5 V of Hepes buffer (25 mM Hepes, pH 7.4; Chaps 0.1%; 5 mM MgCl_2_ and protease inhibitor cocktail Sigma P2714) using the Precellys Evolution (Bertin Technologies). Two cycles of 20 s at 5,500 rpm were performed with the Precellys Homogenizer, and tubes were placed on ice within the two cycles. Levels of TNF-α, IL-1β, IL-6, CXCL1, IL-2, IFNγ, and IL-10 were quantified using multiplex ELISA (Simoa Planar Array Assay Rat Cytokines Panel 1, SP-X, Quanterix). Levels of IGF-1 were quantified using ELISA kits (IGF-1: MG100, R&D Systems).

### Statistical analyses

For all temporal data, permutation statistics were performed using the permutation statistic approach described in Maris and Oostenveld (2007) with Benjamini–Hochberg correction. We used the statistic from Student’s *t* test with 1,000 iterations of permutations with the *t*_mass_ method (mean of the *t*_value_ for each significant cluster). For TA muscle weight data, the normal distribution was assessed by Shapiro–Wilk test and followed with Student’s *t* test. After assessing normal distribution for biochemical assays, we used a one-factor ANOVA with Tukey’s post hoc tests (*α* = 0.05) with Bonferroni’s correction.

## Results

### Cumulative effects of the adaptive sleep response to the muscle injury *plus* sleep extension

First, the objective was to evaluate if the change of the photoperiod (from LD 12:12 to LD 16:8) induces an increase in the sleep time of animals after muscle injury. Based on telemetric EEG/EMG recordings, we analyzed total sleep time (TST) as NREM/REM sleep time for injured and control animals submitted to the LD 16:8 photoperiod (*n* = 8, respectively). These parameters were evaluated for the extended light period (20:00–24:00), for the common dark period (24:00–08:00), for the common light period (08:00–20:00), and per days (08:00–08:00). To accurately discriminate the effect of photoperiod lengthening and muscle injury on sleep, we also compared these data with those obtained for injured animals submitted to LD 12:12 in a previous study (Vanneau et al., 2023). We chose to express sleep results as a percent change from the baseline (established over 3 d prior to Day 0 of injury in conventional LD 12:12 photoperiod); this allows evaluating the sleep profile on the same injured animals subjected either to the LD 12:12 (during baseline) or LD 16:8 (from Day 0) in addition to the comparison between LD 12:12 and LD 16:8 animals.

**Figure 2.**
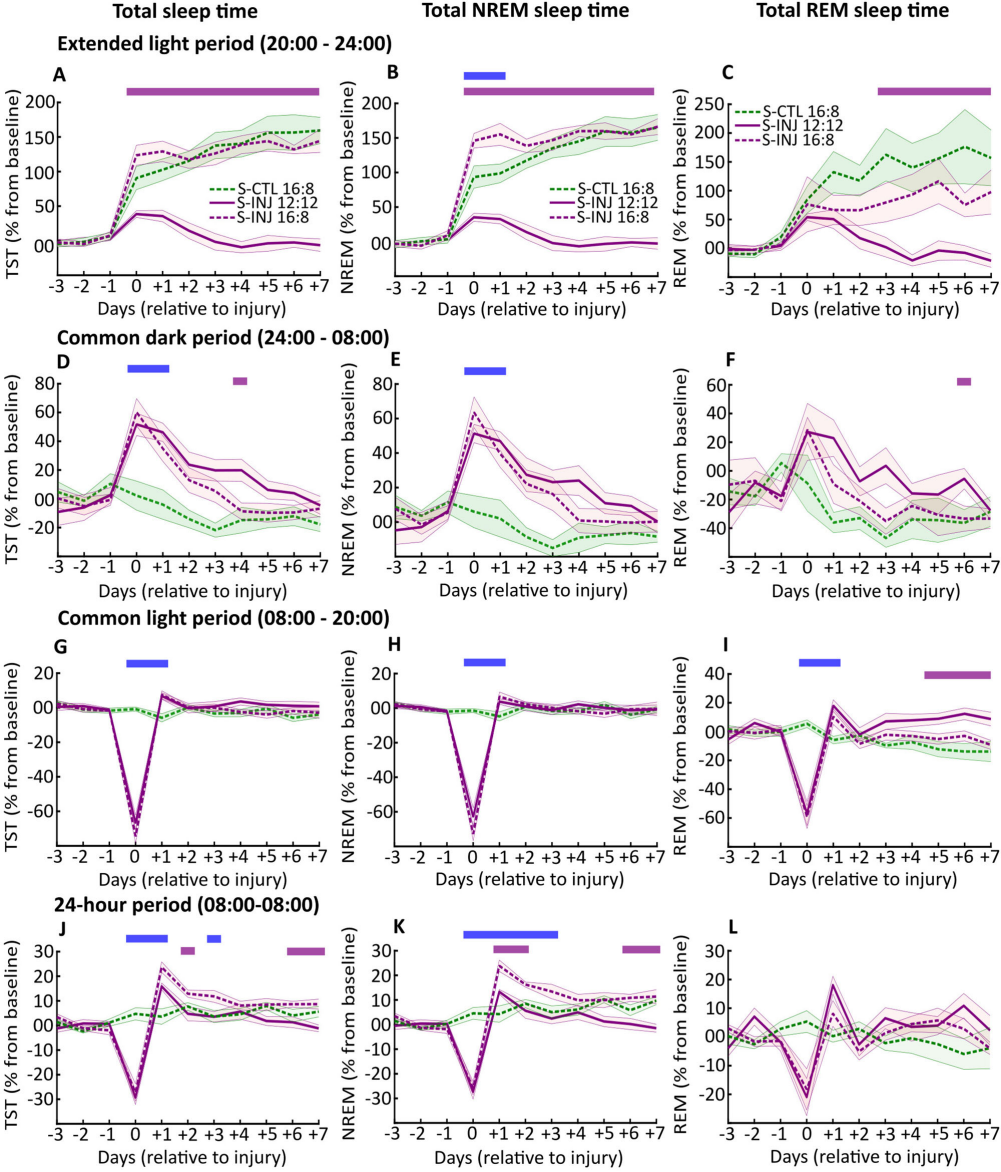
Total and NREM/REM sleep time after photoperiod change. ***A***, Total sleep time (TST) during the 4 h of extended light period (20:00 to 00:00: light period for LD 16:8 group and dark period for 12:12 group) from Day −3 to Day +7 for S-CTL (control animals) LD 16:8 (dashed green line), S-INJ (injured animals) LD 12:12 (solid violet line), and S-INJ LD 16:8 (dashed violet line), expressed in percent change from baseline (average of the corresponding period for Day −3, Day −2 and Day −1). ***B***, ***C***, The same as ***A*** for the total NREM and REM sleep time, respectively. ***D–F***, The same as ***A–C*** for the common light period (from 08:00 to 20:00), ***G–I***, for the common dark period (00:00 to 08:00), and ***J–L*** for the 24-hour period (08:00 to 08:00). Statistical differences (evaluated by permutations statistics; see Materials and Methods) between S-INJ LD 12:12 and S-INJ LD 16:8 are shown in violet rectangle and between S-CTL LD 16:8 and S-INJ LD 16:8 in blue rectangle. SEM are in corresponding shaded area color. See Extended Data Figure 2-1 for corresponding subcutaneous temperature.

A previous study from our laboratory showed that the increase in sleep time in response to LD 16:8 photoperiod in uninjured animals occurred during the 4 h of light lengthening (Sauvet et al., 2018). Consistent with these results, we showed this increase in sleep time in control animals submitted to the LD 16:8 photoperiod during the light extension period (Fig. 2*A*). In addition, injured animals submitted to the LD 16:8 photoperiod benefit from the light extension period from the first day postinjury until the end of the analysis period, with higher TST from Day 0 to Day +7 compared with injured LD 12:12 animals, whereas there is no difference with LD 16:8 control animals, highlighting the effectiveness of the change of the photoperiod to induce sleep extension in injured animals (Fig. 2*A*). This increase in TST for LD 16:8 animals is due to an increase in both NREM and REM sleep time (Fig. 2*B*,*C*). In line with these sleep results, the subcutaneous temperature of S-INJ 16:8 animals is lower during the light extension period than that of S-INJ 12:12 animals (Extended Data Fig. 2-1). Furthermore, there appears to be a cumulative effect of the adaptive sleep response to muscle injury *plus* sleep extension, resulting in higher NREM sleep time for 24 h postinjury for injured LD 16:8 compared with that for control LD 16:8 animals during the light extension period (Fig. 2*B*). Supporting this idea, during the common dark period, there was a similar increase in total and NREM sleep time for 48 h for both injured groups, suggesting an intact adaptive sleep response to muscle injury for injured LD 16:8 animals (Fig. 2*D*,*E*). In agreement with our previous results, there is a significant decrease in total sleep time during the light period on Day 0 for injured animals (Fig. 2*G*). This is a side effect of the analgesic buprenorphine that does not last >24 h (Gauthier et al., 2011; O’Brien et al., 2021; Vanneau et al., 2023). Finally, when considered per days, injured animals submitted to the LD 16:8 photoperiod show a higher total and NREM sleep time compared with both LD 16:8 control and LD 12:12 injured animals (Fig. 2*J*,*K*). The difference with LD 16:8 control animals appear to be due to the adaptive sleep response to muscle injury and the difference with LD 12:12 injured animals to the sleep extension induced by the photoperiod change.

10.1523/ENEURO.0433-23.2023.f2-1 Figure 2-1 **Subcutaneous temperature kinetics injured animals at Day -1 and Day +7 after photoperiod change. A** Hour-by-hour change in the subcutaneous temperature for S-INJ 12:12 animals (red line) and for S-INJ 16:8 animals (blue line) at Day -1 and **B** at Day +7 after the photoperiod change. The light extension period is illustrated by the dotted rectangle (20:00–00:00). Statistical differences (evaluated by permutations statistics, see Methods) between S-INJ LD 12:12 and S-INJ LD 16:8 are shown in brown rectangle. SEM are in corresponding shaded area colour. Download Figure 2-1, EPS file.

Consistent with our previous results, REM sleep time is not subjected to change in response to muscle injury when expressed per days, and surprisingly there is no increase with the change in photoperiod (Fig. 2*L*). Closer examination reveals that the increase during the extended light period is counterbalanced by a decrease during the common light period (Fig. 2*C*,*I*).

### Increased sleep quality during the extended light period

Next, we assessed the sleep quality of the LD 16:8 injured animals. As for sleep quantity, we assessed sleep quality parameters (sleep stability, delta power, and spindle density) for the extended light period, for the common dark period, for the common light period, and per days. One of the components of sleep quality is its stability, represented by the duration of sleep periods (Mavanji et al., 2010). While long sleep bouts (>170 s) are associated with sleep stability in rats, shorts sleep bouts (10–40 s) are associated with fragmented sleep.

**Figure 3.**
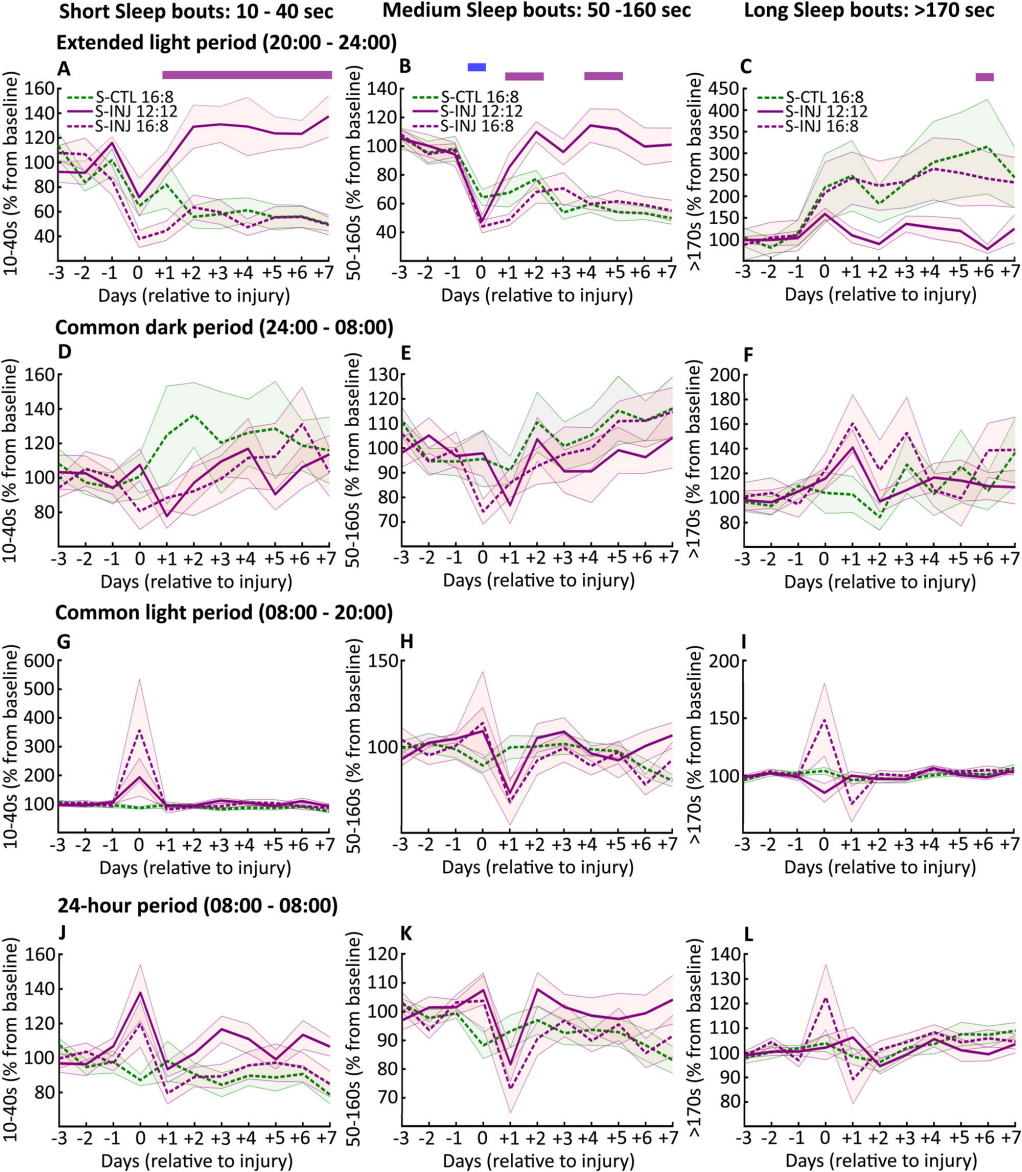
NREM sleep bouts duration after photoperiod change. ***A***, Number of short sleep bouts duration (10–40 s) during the 4 h of extended light period (20:00 to 00:00, light period for LD 16:8 group and dark period for 12:12 group) from Day −3 to Day +7 for S-CTL (control animals) LD 16:8 (dashed green line), S-INJ (injured) LD 12:12 (solid violet line), and S-INJ LD 16:8 (dashed violet line), expressed in percent change from baseline (average of the corresponding period for Day −3, Day −2, and Day −1); ***B*** same as ***A*** for medium sleep bouts duration (50–160 s) and ***C*** for long sleep bouts duration (>170 s). ***D****–**F***, Same as ***A–C*** for the common light period (from 08:00 to 20:00), ***G–I*** for the common dark period (from 00:00 to 08:00), and ***J–L*** for the 24 h period (08:00 to 08:00). Statistical differences (evaluated by permutations statistics, see Materials and Methods) between S-INJ LD 12:12 and S-INJ LD 16:8 are shown in violet rectangle and between S-CTL LD 16:8 and S-INJ LD 16:8 in blue rectangle. SEM are in corresponding shaded area color. See Extended Data Figure 3-1 for REM sleep bouts duration.

**Figure 4.**
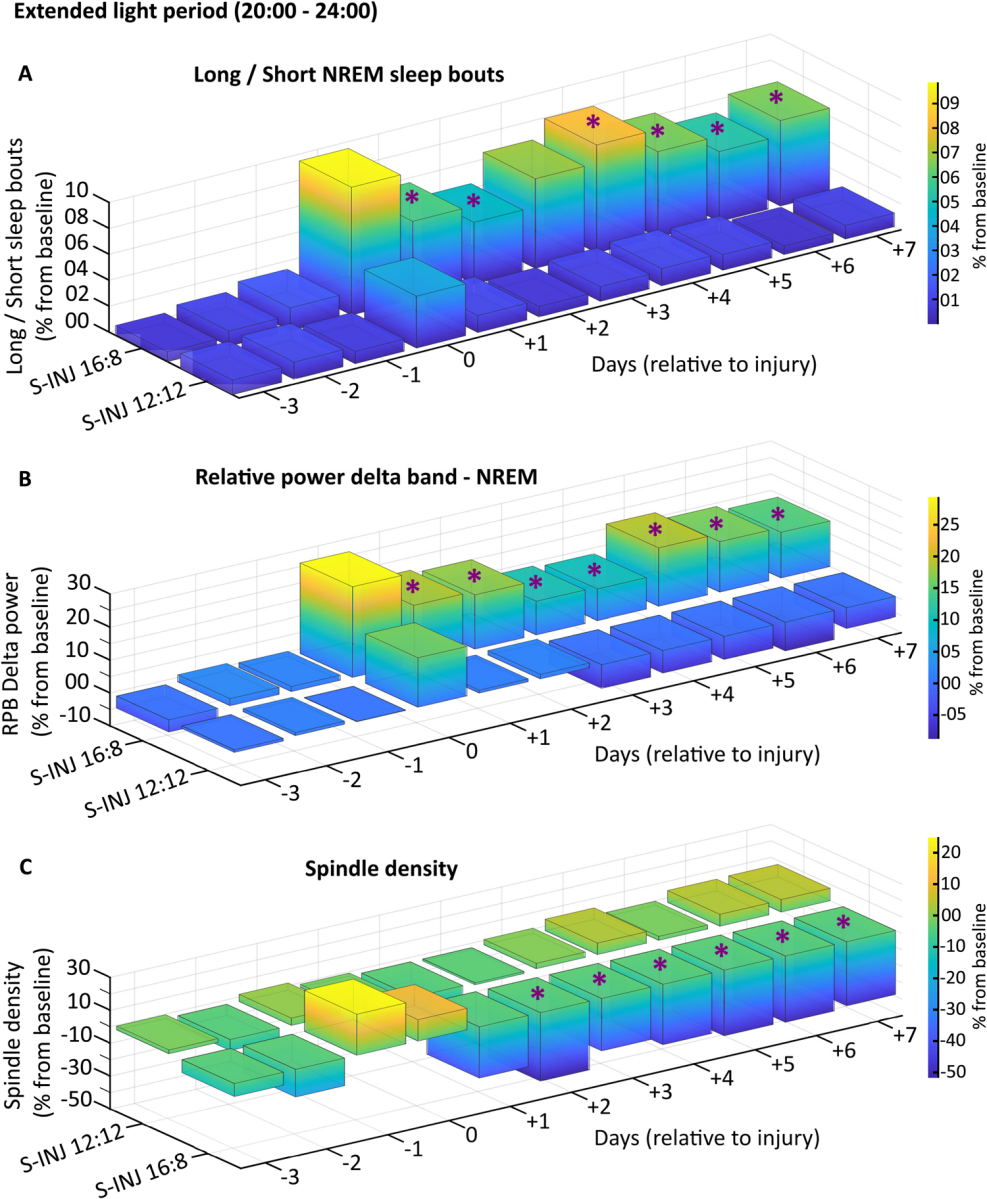
NREM sleep quality during the extended light period (from 20:00 to 00:00) for injured (INJ) animals. ***A***, Ratio of long sleep bouts (>170 s)/short sleep bouts (10–40 s) for the extended light period per hour of NREM sleep expressed in percent change from baseline (average of the corresponding period, i.e., 20:00 to 00:00 for Day −3, Day −2, and Day −1) from Day −3 to Day +7 for S-INJ LD 12:12 and S-INJ LD 16:8 groups. ***B***, Relative power of the delta band (0.25–4 Hz) during NREM sleep for the extended light period expressed in percent change from baseline from Day −3 to Day +7 for S-INJ 12:12 and S-INJ 16:8 groups. ***C***, Spindle density (number of spindle/min of NREM sleep) during the extended light period expressed in percent change from baseline for S-INJ LD 12:12 and S-INJ LD 16:8 groups. Statistical differences (evaluated by permutations statistics; see Materials and Methods) between S-INJ LD 12:12 and S-INJ LD 16:8 are shown with violet asterisk. See Extended Data Figure 4-1 for detailed spectral analysis and Extended Data Table 4-1 along with Extended Data Figure 4-2 for corresponding locomotor activity.

Whether for short, medium, or long NREM sleep episodes, there is no difference when expressed for the common light period, for the common dark period, and per days (Fig. 3*D–L*). However, during the extended light period, the frequency of short sleep episodes is lower, and the frequency of long sleep episodes is higher in LD 16:8 than that in LD 12:12 injured animals (Fig. 3*A*,*C*). This leads to a higher ratio of the frequency of long to short sleep episodes, highlighting greater sleep stability in injured LD 16:8 animals (Fig. 4*A*). For REM sleep, there is no significant change, highlighting an improvement in sleep quality in the LD 16:8 animals (Extended Data Fig. 3-1).

10.1523/ENEURO.0433-23.2023.f3-1 Figure 3-1 **REM sleep bouts duration after photoperiod change. A** Number of short sleep bouts duration (10-40 seconds) during the 4 hours of extended light period (20:00 to 00:00: light period for LD 16:8 group and dark period for 12:12 group) from Day -3 to Day +7 for S-CTL (control animals) LD 16:8 (dashed green line), S-INJ (injured) LD 12:12 (solid violet line) and S-INJ LD 16:8 (dashed violet line), expressed in percent change from baseline (average of the corresponding period for Day -3, Day -2 and Day -1); **B** same as **a** for medium sleep bouts duration (50-160 seconds). **C**, **D**, same as **A**, **B**, and **E** number of long sleep bouts duration (>170 seconds) for the common light period (from 08:00 to 20:00), **F**, **G**, for the common dark period (from 00:00 to 08:00) and **H**, **I**, **J** for the 24-hour period (08:00 to 08:00). Statistical differences (evaluated by permutations statistics, see Methods) between S-INJ LD 12:12 and S-INJ LD 16:8 are shown in violet rectangle and between S-CTL LD 16:8 and S-INJ LD 16:8 in blue rectangle. SEM are in corresponding shaded area colour. Download Figure 3-1, EPS file.

10.1523/ENEURO.0433-23.2023.f4-1 Figure 4-1 **Delta (0.25-4 Hz) and theta (4.25-8.0 Hz) relative power band after photoperiod change. A** Relative power of the theta band in the REM sleep for the extended light period (20:00–00:00) in S-CTL (control animals) LD 16:8 (dashed green line), S-INJ (injured) LD 12:12 (solid violet line) and S-INJ LD 16:8 (dashed violet line), expressed in percent change from baseline from Day -3 to Day +7; **B** Relative power of delta in NREM sleep and **C** for theta in REM sleep during the common light period (08:00–20:00) from Day -3 to Day +7 for S-CTL 16:8 (dashed green line), S-INJ 12:12 (solid violet line) and S-INJ 16:8 (dashed violet line), expressed in percent change from baseline (average of the corresponding period for Day -3, Day -2 and Day -1); **D, E**, the same as **B, C** during the common dark period (00:00–08:00); **F, G**, the same as **B, C** for the 24-hour period (08:00 to 08:00). Statistical differences (evaluated by permutations statistics, see Methods) between S-INJ LD 12:12 and S-INJ LD 16:8 are shown in violet rectangle, between S-CTL LD 16:8 and S-INJ LD 16:8 in blue rectangle. SEM are in corresponding shaded area colour. Download Figure 4-1, EPS file.

10.1523/ENEURO.0433-23.2023.f4-2 Figure 4-2 **Locomotor parameters for injured animals at Day -1 and Day +7 after photoperiod change. A** Hour-by-hour change in the distance covered for S-INJ 12:12 animals (red line) and for S-INJ 16:8 animals (blue line) at Day -1 and **B** at Day +7 after the photoperiod change. The light extension period is illustrated by the dotted rectangle (20:00–00:00). **C**, **D** same as **A**, **B** for the percentage of body in motion. Statistical differences (evaluated by permutations statistics, see Methods) between S-INJ LD 12:12 and S-INJ LD 16:8 are shown in brown rectangle. SEM are in corresponding shaded area colour. Download Figure 4-2, EPS file.

10.1523/ENEURO.0433-23.2023.t4-1 Table 4-1 **Locomotor parameters after the photoperiod change.** #: p<0.05 compared to S-CTL LD 16:8. Three factor ANOVA (Days, photoperiod, and injury) followed by Tuckey post-hoc test. Download Table 4-1, DOCX file.

Another parameter associated with sleep quality is the amount of slow waves (i.e., delta power) during NREM sleep (Vyazovskiy, 2015). Slow-wave sleep is associated with growth hormone (GH) release and protein synthesis (Ramm and Smith, 1990; Van Cauter et al., 2004), and delta power represents the restorative power of sleep (Prinz et al., 1995; Tasali et al., 2008). In parallel with the increase in sleep stability, relative delta power during NREM sleep increases during the extended light period in LD 16:8 compared with that in LD 12:12 injured animals from Day +1 to Day +7 (Fig. 4*B*), whereas there is no change in other periods (Extended Data Fig. 4-1).

We also assessed spindle density, a parameter associated with several functions such as sleep stability (Fernandez and Lüthi, 2020). Surprisingly, the spindle density (number of spindles per min of NREM sleep) during the extended light period is lower for LD 16:8 compared with that for LD 12:12 injured animals from Day +2 to Day +7 (Fig. 4*C*).

To take account of the effects of changes in photoperiod on the animals’ locomotor activity, which plays an important role in sleep regulation, we measured the distance covered and the percentage of body in motion per day by filming them continuously in their living cage. For daily average, the only difference is a higher percentage of body in motion in injured LD 12:12 and LD 16:8 animals compared with that in control LD 16:8 animals on the day of injury induction (Day 0; Extended Data Table 4-1). We carried out a more detailed analysis of locomotor parameters by studying hour-by-hour kinetics on Day −1 and Day +7. The distance covered is lower in 16:8 injured animals compared with that in 12:12 injured animals during the extended light period (Extended Data Fig. 4-2).

### Photoperiod change limits muscle injury-induced mechanical hyperalgesia and induces faster return to baseline paw withdrawal threshold

**Figure 5.**
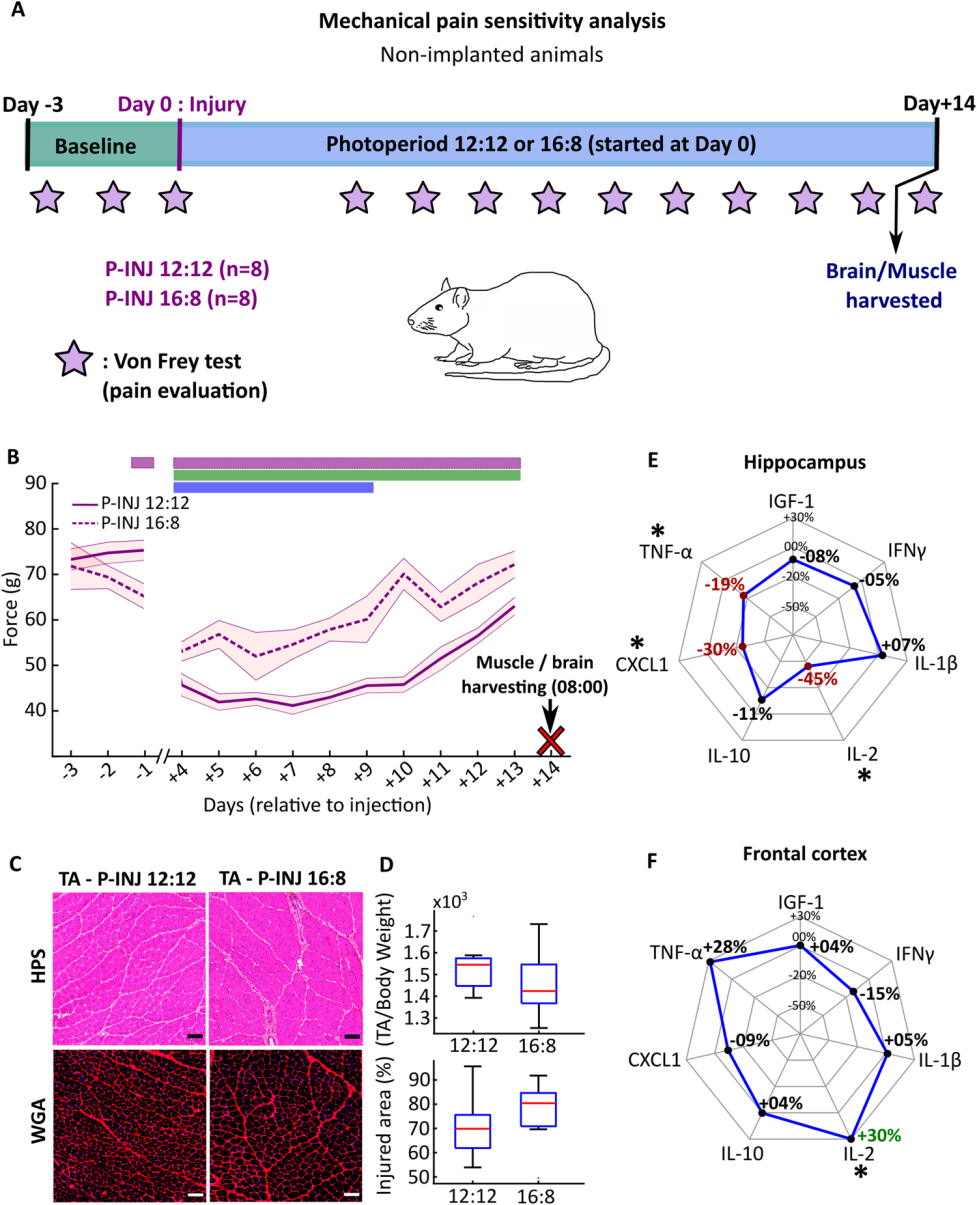
Mechanical hyperalgesia related to muscle injury for EEG-nonimplanted animals after photoperiod change. ***A***, Animals for mechanical hyperalgesia analysis were EEG nonimplanted to avoid potential inflammation. They were separated into two groups (*n* = 8 per group): Pain-(P-)INJ LD 12:12 and P-INJ LD 16:8. Paw withdrawal threshold were assessed from Day −3 to Day −1 and from Day +4 to Day +13 with an electronic Von Frey test (stars) on the injured paw (the right paw). We harvested the TA muscles and the hippocampus and frontal cortex at Day +14. ***B***, Right paw withdrawal thresholds (in grams) during the Von Frey test from Day −4 to Day +13 for P-INJ LD 12:12 animals (solid violet line) and for P-INJ LD 16:8 animals (dashed violet line). Statistical differences (evaluated by permutations statistics; see Materials and Methods) between P-INJ LD 12:12 animals and P-INJ LD 16:8 animals are shown in violet rectangle; between postinjury thresholds and baseline thresholds for P-INJ LD 12:12 in green rectangle and for P-INJ LD 16:8 in blue rectangle. SEM are in corresponding shaded area color. ***C***, Cross section of TA muscle harvested at Day +14 and stained with HPS and WGA immunostaining, respectively, for P-INJ LD 12:12 animal and for P-INJ LD 16:8 animals. ***D***, The ratio TA weight/body weight and the percent of injured area, respectively, for P-INJ LD 12:12 and P-INJ LD 16:8 groups. ***E***, The percent difference ([LD16:8]/[LD12:12]) – 1)*100) between injured animals for protein concentrations in the hippocampus of insulin-like growth factor (IGF)-1, interferon gamma (IFN-γ), interleukin-1 beta (IL-1β), interleukin-2 and -10 (IL-2 and IL-10), C-X-C motif chemokine ligand 1 (CXCL1), and tumor necrosis factor alpha (TNF-α). ***F***, Same as ***E*** in the frontal cortex. See Extended Data Table 5-1 for detailed values. *p < 0.05 [1-factor (photoperiod) ANOVA followed by Tukey’s tests], numbers in dark red indicate significant lower concentration in LD 16:8 compared with that in LD 12:12 injured animals and numbers in green significant higher concentration in LD 16:8 injured animals.

Finally, to assess the effects of the LD 16:8 photoperiod on mechanical sensitivity postinjury, a Von Frey test was applied from Day −3 to Day +13 on EEG-nonimplanted injured animals (Pain-animal's groups) submitted either to the LD 12:12 or the LD 16:8 photoperiod (Fig. 5*A*). For both groups, there is a drop in the paw withdrawal threshold after muscle injury, which indicates mechanical hyperalgesia (Fig. 5*B*). However, the paw withdrawal thresholds are significantly lower for LD 12:12 compared with those for LD 16:8 animals from the first day of measurement after injury (at Day +4 postinjury) to the end of the experiment (at Day +13 postinjury), demonstrating that LD 16:8 limits the mechanical hyperalgesia induced by muscle injury (Fig. 5*B*). Furthermore, the paw withdrawal thresholds are significantly lower from Day +4 to Day +13 postinjury compared with baseline levels for LD 12:12 animals and lower only from Day +4 to Day +9 postinjury compared with baseline threshold for LD 16:8 animals. Thus, LD 16:8 animals recovered their baseline paw withdrawal threshold at Day +10, whereas LD 12:12 animals did not recover it even at Day +13 (Fig. 5*B*).

10.1523/ENEURO.0433-23.2023.t5-1 Table 5-1 **Protein concentrations of sleep regulating molecules in injured animals (see Figure 5).** *: p<0.05 between P-INJ LD 16:8 and P-INJ LD 12:12, Student t test. Download Table 5-1, DOCX file.

At Day +14, animals were killed and we collected the injured TA muscles and brain tissues (hippocampus and frontal cortex). We illustrated the injured TA of the P-INJ LD 12:12 group and the P-INJ LD 16:8 group with HPS staining and WGA immunostaining (Fig. 5*C*), which are used to calculate the percentage of the injured area. Muscle injury parameters (muscle weight and lesion area) are similar between LD 12:12 and LD 16:8 injured animals (Fig. 5*D*). For pro-inflammatory molecules involved in the regulation of pain and sleep in the brain, the concentrations (pg/mg) of TNF-α, IL-2, and CXCL1 in the hippocampus are lower in LD 16:8 compared with those in LD 12:12 injured animals, and there is no difference for IGF-1, IFNγ, IL-1β, and IL-10 (Fig. 5*E*). In the frontal cortex, the only difference is a higher concentration of IL-2 in P-INJ LD 16:8 compared with that in P-INJ LD 12:12 animals (Fig. 5*F*; detailed values are in Extended Data Table 5-2).

## Discussion

The present study investigated the efficiency of photoperiod lengthening (a rat model of sleep extension) to increase sleep duration and sleep quality in muscle-injured rats and assessed the effects on mechanical hyperalgesia related to muscle injury using the Von Frey test. We carried out this study into two parts. Sleep parameters were assessed on EEG-implanted animals after myotoxic skeletal muscle injury, and muscle injury-induced mechanical hyperalgesia on nontelemetry-implanted animals.

Our first objective was therefore to evaluate whether lengthening the conventional LD 12:12 photoperiod to a LD 16:8 photoperiod increased sleep time in rats after muscle injury, as we have previously shown in uninjured animals (Sauvet et al., 2018). In this previous study, the total sleep time (TST) and NREM sleep time increased significantly during the 4 h of light lengthening (from 20:00 to 24:00). In the herein presented results, for this same period of light lengthening, the TST and NREM sleep time of LD 16:8 injured animals are higher than those of LD 12:12 injured animals from Day 0 to Day +7, and REM sleep time was higher from Day +3 to Day +7. This higher TST was associated with lower subcutaneous temperature and locomotor activity during the light lengthening period of LD 16:8 injured animals compared with that of LD 12:12 injured animals. We also confirmed that TST was higher in LD 16:8 versus LD 12:12 noninjured [i.e., control (S-CTL) 12:12, not shown in the figures] as previously described (Sauvet et al., 2018), and we found no statistical difference for TST between injured and control LD 16:8 animals. Furthermore, during the extended light period (from 20:00 to 24:00), the NREM sleep time of LD 16:8 injured animals was higher than that of LD 16:8 control animals for 24 h postinjury. In addition, during the common dark period, we observed a similar increase in NREM sleep time (and in total sleep time) for LD 12:12 and LD 16:8 injured animals for 24 h postinjury. The latter two observations underscore the adaptive sleep response to muscle injury for LD 16:8 injured animals as shown previously (Vanneau et al., 2023), additionally pointing a cumulative effect with photoperiod-induced sleep extension. This could be surprising given that the extended light period occurs just before the dark period during which the adaptive sleep response to injury is observed. Thus, although injured animals had an increase in NREM sleep time during the extended light period, they still have an increase during the subsequent dark period, which may reflect the high sleep need of injured animals.

Nonetheless, REM sleep time was lower in LD 16:8 than in LD 12:12 injured animals during the common 12 h light period (i.e., the period preceding the 4 h light lengthening period) and also statistically at Day +6 during the common 8 h dark period (i.e., the period following after the light lengthening period), suggesting specific homeostatic regulation of the daily amount of REM sleep (Barbato and Wehr, 1998; Park and Weber, 2020). Indeed, the specific decrease in REM sleep time during the common light period may counterbalance the increase in REM sleep during the extended light period.

Furthermore, besides the increases in sleep quantity during the extended light period in LD 16:8 compared with those in 12:12 injured animals, there was both a decrease of short (10–40 s) and an increase in long (>170 s) NREM sleep period frequency, resulting in more stable sleep in LD 16:8 injured animals (Gall et al., 2008; Stephenson et al., 2012). Associated with this greater sleep stability, we also observed higher relative delta power during NREM sleep, which is associated with better restorative sleep (Decker et al., 2009; Gorlova et al., 2019). These findings of increased sleep quantity and quality during the extended light period persist throughout the duration of the EEG recordings. During the extended light period, there is also a lower density of spindles for LD 16:8 injured animals compared with LD 12:12 injured animals, which may be questionable. Data in the literature indicate that during NREM sleep, delta power and sigma power (9–16 Hz) are oppositely regulated: as sleep duration progresses, delta power decreases and sigma power increases, resulting in higher spindle density as sleep duration progresses (Fernandez and Lüthi, 2020). We can therefore suggest that lower spindle density during the extended light period for LD 16:8 injured animals could be related to the increase in delta power during the same period.

Finally, we evaluated the effect of photoperiod change on muscle injury-induced mechanical hyperalgesia in non-EEG–implanted animals. Indeed, most studies on the potential positive role of sleep on pain sensitivity have been carried out in humans, and to our knowledge there are no animal studies. Extended bedtime reduces pain sensitivity in mildly sleepy healthy adults (Roehrs et al., 2012), and sleep extension increases pain tolerance but does not affect the pain threshold in normally sleeping individuals (Simonelli et al., 2019). Our results firstly showed that the paw withdrawal thresholds measured at baseline are similar to those described in literature (Ferrier et al., 2016). Secondly, at the first measurement 4 d after muscle injury induction, decreased paw withdrawal thresholds were observed for both LD 12:12 and LD 16:8 animals, indicating a mechanical hyperalgesia due to injury. However, paw withdrawal thresholds were significantly lower for LD 12:12 compared with those for LD 16:8 injured animals, from Day +4 to the end of the experiment at Day +13, indicating that the change in photoperiod limits muscle injury-induced mechanical hyperalgesia. In addition, all the paw withdrawal thresholds measured for LD 12:12 injured animals after the injury induction (from Day +4 to Day +13) are significantly lower compared with baseline thresholds (from Day −3 to Day −1), whereas there is no difference between thresholds measured after Day +9 compared with baseline values for LD 16:8 injured animals. Therefore, the photoperiod lengthening also allows faster recovery of baseline paw withdrawal threshold. To our knowledge, our study is thus the first to suggest that sleep extension induced by photoperiod lengthening is beneficial for muscle injury-induced mechanical hyperalgesia in animals. Importantly, we chose to assess pain in nontelemetry-implanted animals, so as not to induce inflammation unrelated to the muscle injury. The sleep time of these animals was therefore not evaluated. Although these animals were in the same conditions as the telemetry-implanted animals, for which we demonstrated the effectiveness of sleep extension, this is a limitation of the mechanical sensitivity result.

A factor potentially involved in the beneficial effect of photoperiod change on mechanical sensitivity could be a difference in locomotor activity between animals injured in LD 16:8 and LD 12:12, as it has been shown that immobilizing a muscle in the first few days after injury is beneficial for muscle healing (Järvinen et al., 2005, 2007). Consistent with a previous study (Sauvet et al., 2018), the locomotor activity of injured (and telemetry implanted) LD16:8 animals is lower than that of injured LD 12:12 animals during the light extension period (Extended Data Fig. 4-2). However, the daily average of distance covered, or percentage of body in motion, is not different between these two groups of animals (Extended Data Table 4-1).

The next challenge was to understand the mechanism by which photoperiod lengthening reduces mechanical hyperalgesia after muscle injury. To answer this, we analyzed histological and biochemical parameters on tissues harvested 14 d after injury induction. First, the area of injury as the muscle weight was not different between LD 12:12 and LD 16:8 animals, suggesting that the difference in mechanical sensitivity, at least at Day +13, does not come from a difference in the size of the muscle injury. Second, we observed lower concentrations of pro-inflammatory cytokines, TNF-α, IL-2, and CXCL1, in the hippocampus, while the neurotrophic factor IGF-1 is not statistically different. Several studies have shown that chronic pain is associated with abnormalities in hippocampal functioning and structure (e.g., behavior, synaptic plasticity, and neurogenesis) that may be related to a local increase in inflammatory and/or a decrease in neurotrophic factors (Mutso et al., 2012; Mokhtari et al., 2019). Furthermore, sleep and pain are closely interconnected, and sleep deprivation increases inflammatory biomarkers (such as TNF-α) in the hippocampus of animals (Chennaoui et al., 2015) and in the plasma of healthy subjects in association with increased pain experience (Haack 2007; Chennaoui et al., 2015; Faraut et al., 2015; Nijs et al., 2017; Wadhwa et al., 2017). In view of our results, we might suggest that sleep extension induced by photoperiod lengthening produces an anti-inflammatory effect at the hippocampus level that could induce resilience to muscle injury-induced mechanical hyperalgesia (Nijs et al., 2017; Vasic and Schmidt, 2017). However, the limitation of this result is that we do not have biochemical data on the two brain structures throughout the pain assessment. Additional studies are needed to define whether decreased pro-inflammatory cytokines in the hippocampus is involved in the beneficial effects of sleep extension on muscle injury-induced mechanical hyperalgesia.

In conclusion, our results demonstrate the efficiency of photoperiod lengthening to induce sleep extension in animals after skeletal muscle injury. Furthermore, they show a cumulative effect of sleep extension-related to photoperiod lengthening *plus* the adaptive sleep response to muscle injury, revealing a high need for sleep after muscle injury. This is corroborated by the increase in sleep quality during the extended light period for injured animals. In addition, the photoperiod modification limits the mechanical hyperalgesia induced by muscle injury and enabled faster recovery of baseline thresholds, which was associated with a decrease in pro-inflammatory markers in the hippocampus. Future studies should focus on the effects of sleep extension on the molecular processes of postinjury muscle repair through recovery of muscle function.

## Synthesis

Reviewing Editor: Jibran Khokhar, Western University Department of Anatomy and Cell Biology Decisions are customarily a result of the Reviewing Editor and the peer reviewers coming together and discussing their recommendations until a consensus is reached. When revisions are invited, a fact-based synthesis statement explaining their decision and outlining what is needed to prepare a revision will be listed below. The following reviewer(s) agreed to reveal their identity: NONE. Note: If this manuscript was transferred from JNeurosci and a decision was made to accept the manuscript without peer review, a brief statement to this effect will instead be what is listed below.

This paper explored the effects of photoperiod lengthening on sleep duration after muscle injury. Photoperiod lengthening helped reduce hyperalgesia and inflammation and could be a potential non-pharmacological tool injury-related hyperalgesia. The authors address the reviewers comments thoroughly.
