# Supplementary material for: Beneficial Effects of Photoperiod Lengthening on Sleep Characteristics and Mechanical Hyperalgesia in Injured Rats
Source: eNeuro. 2024 Mar 1;11(3):ENEURO.0433-23.2023. doi: 10.1523/ENEURO.0433-23.2023 (PMC10921263; doi:10.1523/ENEURO.0433-23.2023)
Supplement: Table 4-1 — Locomotor parameters after the photoperiod change. #: p<0.05 compared to S-CTL LD 16:8. Three factor ANOVA (Days, photoperiod, and injury) followed by Tuckey post-hoc test. Download Table 4-1, DOCX file. [file eneuro-11-ENEURO.0433-23.2023-s001.docx]

|  | Day -1 | Day 0 | Day +1 | Day +2 | Day +3 | Day +4 | Day +5 | Day +6 | Day +7 | Day +8 |
| --- | --- | --- | --- | --- | --- | --- | --- | --- | --- | --- |
| **Distance covered (cm)** | | | | | | | | | | |
| S-CTL 16:8 | 864 ±293 | 830 ±292 | 805 ±267 | 818 ±279 | 836 ±275 | 836 ±279 | 863 ±275 | 900 ±306 | 826 ±266 | 745 ±247 |
| S-INJ 12:12 | 989 ±324 | 1228 ±315 | 740 ±239 | 781 ±231 | 850 ±248 | 875 ±276 | 882 ±280 | 914 ±299 | 941 ±291 | 896 ±282 |
| S-INJ 16:8 | 729 ±232 | 1120 ±322 | 578 ±196 | 626 ±205 | 627 ±203 | 649 ±215 | 685 ±230 | 717 ±256 | 706 ±250 | 583 ±192 |
| **Percentage of body in motion (%)** | | | | | | | | | | |
| S-CTL 16:8 | 19 ±0.09 | **16 ±0.06** | 15 ±0.05 | 16 ±0.06 | 16 ±0.05 | 16 ±0.05 | 16 ±0.05 | 18 ±0.07 | 16 ±0.05 | 15 ±0.05 |
| S-INJ 12:12 | 19 ±0.07 | **27 ±0.07#** | 13 ±0.04 | 15 ±0.04 | 16 ±0.04 | 16 ±0.05 | 16 ±0.05 | 16 ±0.05 | 22 ±0.07 | 21 ±0.07 |
| S-INJ 16:8 | 17 ±0.05 | **26 ±0.07#** | 15 ±0.05 | 16 ±0.05 | 15 ±0.04 | 17 ±0.05 | 16 ±0.05 | 16 ±0.06 | 16 ±0.05 | 13 ±0.04 |
