# Supplementary material for: Beneficial Effects of Photoperiod Lengthening on Sleep Characteristics and Mechanical Hyperalgesia in Injured Rats
Source: eNeuro. 2024 Mar 1;11(3):ENEURO.0433-23.2023. doi: 10.1523/ENEURO.0433-23.2023 (PMC10921263; doi:10.1523/ENEURO.0433-23.2023)
Supplement: Table 5-1 — Protein concentrations of sleep regulating molecules in injured animals (see Figure 5). *: p<0.05 between P-INJ LD 16:8 and P-INJ LD 12:12, Student t test. Download Table 5-1, DOCX file. [file eneuro-11-ENEURO.0433-23.2023-s002.docx]

|  | IGF-1 (pg/mg) | IFNγ  (pg/mg) | IL-1β  (pg/mg) | Il-2  (pg/mg) | IL-10  (pg/mg) | CXCL1  (pg/mg) | TNF-α  (pg/mg) |
| --- | --- | --- | --- | --- | --- | --- | --- |
| **Hippocampus** | | | | | | | |
| P-INJ 12:12 | 49.62  ±3.99 | 7.75  ±0.48 | 0.90  ±0.02 | **0.95**  **±0.04** | 0.80  ±0.04 | **2.46**  **±0.13** | **3.00**  **±0.15** |
| P-INJ 16:8 | 45.75  ±4.30 | 7.35  ±0.48 | 0.96  ±0.04 | **0.52**  **±0.05*** | 0.72  ±0.05 | **1.73**  **±0.19*** | **2.42**  **±0.18*** |
| **Frontal Cortex** | | | | | | | |
| P-INJ 12:12 | 77.10  ±4.12 | 6.01  ±0.28 | 0.81  ±0.03 | **0.36**  **±0.02** | 0.62  ±0.02 | 0.40  ±0.02 | 1.81  ±0.16 |
| P-INJ 16:8 | 80.22  ±4.11 | 5.12  ±0.48 | 0.85  ±0.02 | **0.47**  **±0.03*** | 0.65  ±0.02 | 0.36  ±0.03 | 2.33  ±0.24 |
